# Quantitative Acetylome Analysis of Soft Wheat Seeds during Artificial Ageing

**DOI:** 10.3390/foods11223611

**Published:** 2022-11-12

**Authors:** Liuke Liang, Aowen Xie, Haojie Yang, Na Li, Ping’an Ma, Shan Wei, Shuaibing Zhang, Yangyong Lv, Yuansen Hu

**Affiliations:** 1 College of Biological Engineering, Henan University of Technology, Zhengzhou 450001, China; 2 College of Life Science and Technology, Huazhong Agricultural University, Wuhan 430070, China

**Keywords:** wheat seeds, lysine acetylation, artificial ageing, proteomics

## Abstract

Lysine acetylation (Kac) is a protein post-translational modification (PTM) widely found in plants that plays vital roles in metabolic pathways. Although seed germination and development are regulated by Kac, its potential function in seed ageing remains to be investigated. Our preliminary study demonstrated that Kac levels were altered during wheat seed artificial ageing. However, its specific role in this process still needs to be elucidated. Here, we performed quantitative acetylation proteomics analysis of soft wheat seeds with different germination rates during artificial ageing. A total of 175 acetylation proteins and 255 acetylation modification sites were remarkably changed. The differentially acetylated proteins were enriched in metabolism; response to harsh intracellular environment, such as ROS; protein storage and processing. Notably, expression, point mutation to mimic Kac by K to Q mutation at K80 and K138, protein purification and enzyme activity detection revealed that the Kac of ROS-scavenging glutathione transferase attenuated its activity, indicating that the defense ability of wheat seeds to stress gradually diminished, and the ageing process was inevitable. Collectively, our data provide a basis for further understanding the roles of Kac in seed ageing and might aid in the development of new techniques to prolong seed viability and food quality.

## 1. Introduction

Wheat (*Triticum aestivum* L.) is a widely cultivated, adaptable and high-yield cereal crop with an annual harvest of over 600 million tons each year. It is commonly used for human food owing to it being a rich source of amino acids, minerals and dietary fiber and other nutrients [1]. However, the seed viability and edible quality of post-harvest wheat inevitably decline during long-term storage, which has a serious impact on agricultural production and food quality [2,3]. Therefore, an in-depth elucidation of the ageing process of wheat during storage has guiding importance to develop effective measures to delay seed ageing and prolong its storage period.

Ageing is a common natural phenomenon during wheat seed storage accompanied with several physiological and biochemical indicators, such as the increase of free fatty acids, malondialdehyde (MDA) content and other toxic cell metabolites, and the remarkable decrease of superoxide free radicals in the later storage period [3]. Many factors play vital roles in wheat grain ageing, including lipid peroxidation; loss of membrane integrity; the inability of cells to scavenge radicals; and the degradation of proteins, nucleic acids and lipids; amongst which, the accumulation of reactive oxygen species (ROS) is one of the major factors [4,5]. The inner antioxidant system can eliminate ROS to combat and balance the redox hemostasis in plants and is essential for the maintenance of seed viability. The enzymes include superoxide dismutase, catalase and peroxidase [5]. Additionally, glutathione transferases (GSTs) and glutaredoxins can also utilize glutathione to remove ROS and maintain protein integrity [6]. One vital function of GSTs is to remove ROS, including superoxide radicals, hydroxyl radicals and hydrogen peroxide, which is important in cellular protection against oxidative stress [7,8].

Artificial ageing is often employed to mimic natural ageing, and proteomic analysis is carried out to investigate changes in physiological and biochemical states during this process [9]. Previous studies showed that the differential proteins in wheat seeds during artificial ageing are involved in nutrient storage, metabolism-related enzyme activity, energy supply and defense responses to stress [10,11]. Furthermore, recent studies demonstrated that the acetylation of metabolism-related enzymes, such as pyruvate kinase, glyceraldehyde-3-phosphate dehydrogenase and pyruvate dehydrogenase, functions in metabolism via regulating protein stability, enzyme activity and controlling protein-protein interactions, which play important roles in seed germination and development [12]. Our preliminary results indicated that the acetylation level fluctuates during the artificial ageing process, and acetylation modification is correlated with wheat seed ageing, which needs to be further elucidated.

In this work, the role of protein acetylation modification in wheat seed ageing was studied using the quantitative acetylome of soft cultivars ‘Yangmai 15’ with different germination rates (97% and 6%) that were treated with artificial ageing. The results showed that the differentially lysine-acetylated proteins preferentially target metabolism and energy, protein storage, enzyme activity regulation, stress response, signal transduction and other functions. Notably, point mutation mimicking lysine acetylation (Kac) at the K80 and K138 residues of GST affected its enzymatic activity, indicating that the Kac of this enzyme is involved in the ageing process of wheat grain during storage. The results provide new insights to further study PTM during the wheat ageing process.

## 2. Materials and Methods

### 2.1. Wheat Seeds and Artificial Ageing Process

The seeds of soft wheat variety ‘Yangmai 15’ selected in this study were purchased from Henan Academy of Agricultural Sciences (Zhengzhou, China), and the artificial ageing method was referred from our previous research [10]. The process is as follows. Artificial ageing of wheat seeds was performed at 45 ± 1 °C and 50% relative humidity, and the viability of wheat seeds was determined regularly. Wheat tissue samples with germination rates of 97% and 6% (named YM97 and YM6, respectively) were collected for the quantitative proteomic analysis of acetylation modifications.

### 2.2. Protein Extraction and Western Blot Analysis

The YM97 and YM6 wheat seed samples were ground to a powder by adding liquid nitrogen using a pre-cooled mortar. Sonic lysis was performed by adding four volumes of phenol extraction buffer (containing 50 mM N-acetylmuramic acid, 2 mM EDTA, 10 mM disthithreitol, 3 μM tryptic soy agar and 1% protease inhibitors). The lysate was centrifuged, the supernatant was precipitated, and the pellet was washed and finally dissolved in 8 M urea as previously described [11]. Western blot analysis was carried out to detect protein acetylation modification levels. The YM97 and YM6 proteins were separated by 12% polypropylene gel, transferred to polyvinylidene fluoride membrane and blocked by bovine serum albumin at 25 °C for 1 h. The samples were incubated with anti-acetyllysine antibody (1:1000 dilution) and secondary antibody (goat anti-mouse IgG, 1:5000 dilution) and then detected by exposure.

### 2.3. Trypsin Digestion, TMT Labelling, Fractionation Process, Enrichment of Lys-Acetylated Peptides, LC-MS Analysis and Database Searches

After the samples were digested by trypsin, the peptides were labelled following the instructions of the tandem mass tag (TMT) kit (Thermo Fisher Scientific, Waltham, MA, USA), then desalted and dried by vacuum freezing. The TMT-labeled peptides were separated by a Thermo Betasil C18 column (Thermo Fisher Scientific, USA). Briefly, the peptides were separated into 60 fractions, combined into four fractions and dried using vacuum centrifugation. The peptide was dissolved in immunoprecipitation (IP) buffer solution and centrifuged. The supernatant was gently shaken with pre-washed acetyl resin at 4 °C overnight. The resin was washed with IP buffer solution, deionized water and 0.1% trifluoroacetic sequentially. The eluent was collected, vacuum-frozen, pumped, desalted and then supplied for liquid mass spectrometry. The peptides were dissolved by liquid chromatographic mobile phase A (0.1% formic acid and 2% acetonitrile) and separated by ultra-high-performance liquid chromatography according to a previous study [13]. The separated peptides were injected into a nanospray ionization source for ionization and then analyzed by mass spectrometry (Q Exactive^TM^ Plus) (Thermo Fisher Scientific, USA). Finally, data were searched using Maxquant (v1.5.2.8) with the reference database (Uniprot Wheat, 116638 sequences) (http://www.ebi.ac.uk/Uniprot/8/8/2022 (accessed on 20 October 2022)) [14], and the reverse decoy database was employed to calculate the false positive rate caused by random matches [15]. 

### 2.4. Bioinformatics Analysis

Motif-X (Schwartz and Gygi, Cambridge, MA, USA) was used to analyze the characteristics of the modified sites. Gene Ontology (GO) annotations were obtained using the UniProt-GOA database (http://www.ebi.ac.uk/GOA/8/8/2022 (accessed on 20 October 2022)) [16]. Pathway enrichment analysis was performed using the Kyoto Encyclopaedia of Genes and Genomes (KEGG) database (http://www.ebi.ac.uk/KEGG/8/8/2022 (accessed on 20 October 2022)) [17]. InterPro database (http://www.ebi.ac.uk/interpro/8/8/2022 (accessed on 20 October 2022)) was employed to investigate the enrichment of the functional domains of differentially modified proteins [18]. Protein interaction network analysis was performed using Cytoscape software 3.7.0 (Schwikowski and Ideker, Seattle, WA, USA).

### 2.5. RNA Extraction, cDNA Synthesis, Cloning of GST Gene and Construction of Point Mutation Expression Vectors

The total RNA of wheat was extracted using Trizol Kit (Takara, Dalian, China) to clone the open reading frame of GST. The total RNA was reverse-transcribed according to the instructions of the PrimeScript™ RT Reagent Kit (Takara, Dalian, China) with gDNA Eraser to complete the synthesis of cDNA strands and stored at −20 °C.

The GST gene was amplified using primers GST-F (AGCGAATTCATGGCTCCGGTGAAGCTG) and GST-R (TAAATATGCGGCCGCTGGCTTCATCATGCGCGCCA) using the cDNA obtained by reverse transcription as the template. The pET-28a vector was linearized by BamHI and EcoRI double enzyme digestion. The target fragment was ligated into the PET-28a vector to generate native GST without mutations. Lysine was mutated to Q to mimic GST acetylation according to previous research [19]. The Quik-Change Site-directed Mutagenesis Kit (Vazyme, Nanjing, China) was employed to generate site-mutated plasmids using primers GST-80Q-1/2 (CAGCTCTGGCTGGTTCTTGCGG/CCGCAAGAACCAGCCAGAGCTG) and GST-138Q-1/2 (GTTCTTGATCTGAACAAGGTTGTCG/CGACAACCTTGTTCAGATCAAGAAC). The plasmids with GST site mutations (GST-80Q and GST-138Q) were transformed into Escherichia coli host strain DH5α, and the positive transformants were confirmed using DNA sequencing.

### 2.6. GST Expression and Purification and Measurement of Its Activity

The recombinant plasmid with correct sequencing was transformed into the expressing strain *E. coli* BL21. Single colonies were inoculated in LB medium with a final kanamycin concentration of 50 mg/mL at 37 °C at 200 r/min until the OD_600_ was 0.6. Isopropyl thiogalactoside (final concentration = 0.5 mM/100 μL) was added to induce protein expression at 16 °C. Then, a 1 mL of the culture was collected and centrifuged at 12,000 r/min for 2 min, and the strain was collected. The loading buffer was added, mixed, boiled for 5 min, centrifuged and analyzed using sodium dodecyl sulphate-polyacrylamide electrophoresis (SDS-PAGE).

The target protein was purified by Ni^2+^-NTA affinity chromatography. The cultured cells were centrifuged at 10,000 r/min for 10 min, quickly frozen in liquid nitrogen and stored at −80 °C. Then, the cells were suspended with buffer A (500 mM NaCl, 20 mM Tris-HCl, pH 8.0) until the final volume was 13 mL, and ultrasonic crushing was performed for 30 min. The bound proteins were eluted using buffer A containing 0, 20, 40, 80 and 300 mM imidazole. A 40 μL fraction of the eluent was collected for every elution and identified by SDS-PAGE.

The enzyme activities of GST, GST-80Q and GST-138Q were measured following the instructions of the GST Activity Detection Kit (Solarbi, Beijing, China). Briefly, the sample and reagent 1 were homogenized in an ice bath at a ratio of 1:10 and centrifuged at 8000× *g* at 4 °C for 10 min. The supernatant was placed on ice for testing. The reagents (100 μL supernatant, 100 μL reagent 1, 900 μL reagent 2 and 100 μL reagent 3) were mixed. The absorbance measured at 340 nm was denoted as *A*_3_, and the absorbance measured after a water bath at 25 °C for 5 min was denoted as *A*_4_. The corresponding absorbance values of the blank group were denoted as *A*_1_ and *A*_2_, respectively. GST activity was calculated according to the formula:

$$\mathrm{GST}\;\left(U/\mathrm{mg}\right)=0.23\times \left[\left(A_{4}-A_{3}\right)-\left(A_{2}-A_{1}\right)\right]÷C_{\mathrm{pr}}$$

where:

*A*_1_—Absorbance of the blank group sample;

*A*_2_—Absorbance of blank group sample after water bath;

*A*_3_—Absorbance of experimental group sample;

*A*_4_—Absorbance of experimental group sample after water bath;

*C*_pr_—Protein concentration of supernatant (mg/mL).

## 3. Results

### 3.1. Detection of Acetylated Proteins and Motif Analysis of Modification Sites

**Figure 1:**
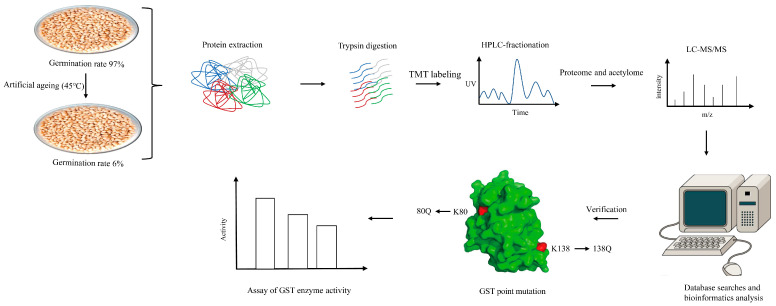
Process of quantitative acetylome analysis of wheat seeds.

**Figure 2:**
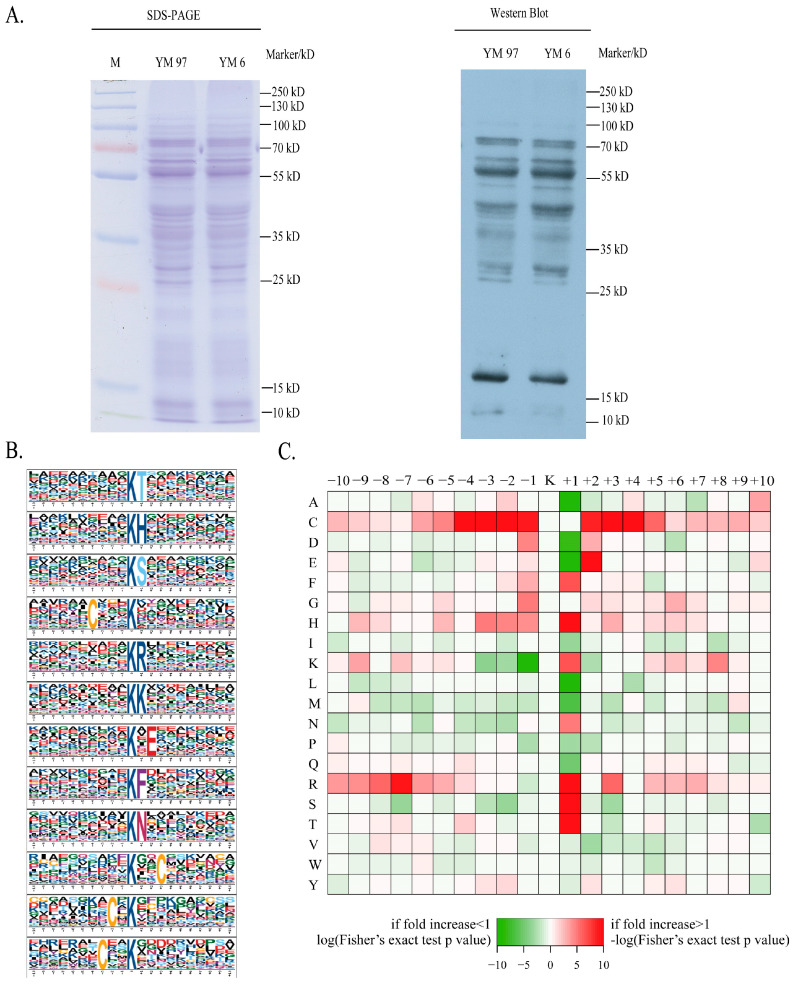
Detection and motif analysis of Kac in wheat seed ageing process. (**A**) Identification of Kac in wheat by pan anti-acetyllysine antibody. (**B**) Acetylation sequence motifs around Kac sites. (**C**) Heat map distribution of amino acids near the acetylated sites.

The main aim of this study was to reveal the underlying mechanisms regulating wheat seed artificial ageing at the acetylome level. The initial germination rate of wheat seeds was 97%, and the germination rate after artificial ageing was 6% according to our previous research [10]. Samples with germination rates of 97% and 6% were selected for quantitative acetylome analysis. Subsequently, verification of the Kac in enzymes involved in artificial ageing was performed. The point mutation to mimic lysine acetylation and enzyme activity detection of the differentially acetylated protein glutathione transferases were carried out to elucidate its potential roles in artificial ageing. The overall process of the quantification of acetylated proteome is shown in Figure 1. The SDS-PAGE analysis results demonstrated that the protein bands were clear and uniform without degradation. Then, the proteins were detected by Western blot using the Kac pan-antibody. The results showed that the Kac was detected in the two samples but varied in the different molecular weights of YM97 and YM6 protein samples, which suggests that the Kac might function during wheat seed ageing (Figure 2A).

The 10 amino acid sequences upstream and downstream of all acetylation sites were statistically analyzed, and the position-specific amino acid frequencies at both ends of the acetylation sites were calculated using motif-x to characterize the motif properties of the acetylation sites. These sites matched 11 conserved motifs, of which the top three were XXXXXXXXXX Kac TXXXXXXXXX, XXXXXXXXXX KacH XXXXXXXXX and XXXXXXXXXX KacS XXXXXXXXX (Kac represents acetylated lysine, and X represents arbitrary amino acids; Figure 2B). Our results showed that KacH and KacS motifs were enriched in other species, such as Trichinella spiralis [20], Aspergillus flavus [13] and rice [21]. The results indicate its conservation in different species.

The motif enrichment heat map results showed that cysteine (C) enrichment was high at the −4, −3, −2, −1, +2, +3 and +4 sites, and the occurrence of arginine (R) was enriched at the −7 site downstream of the modification site. Most of the conserved residues (histidine (H), R, serine (S), threonine (T) and E) were located at +1 and +2 upstream of the modification sites. This result was similar to those observed in T. spiralis [20] and rice [21]. However, alanine (A), aspartic acid (D), glutamate (E), leucine (L) and lysine (K) appeared less frequently at the +1 and −1 positions. Additionally, our research results showed that cysteine (C) was the most conserved amino acid in the upstream and downstream regions, followed by arginine (R) (Figure 2C).

### 3.2. Identification of Modification Sites and Proteins

For the quality control for mass spectrometry, the tolerance of peptides and length distribution met the requirements, and the standard deviation coefficient (RSD) was less than 0.1, indicating that the samples were qualified for the following analysis (Figure S1A–C). A total of 1663 acetylation sites were detected on 892 proteins, of which 1526 sites on 830 proteins were quantitatively analyzed. The threshold of significant up-regulation was more than 1.3 times, and that of down-regulation was less than 1/1.3. A total of 175 differentially acetylated proteins, including 84 up-regulated and 91 down-regulated acetylated proteins, were characterized in the YM6 versus YM97 comparison. Moreover, 255 differentially acetylated sites were confirmed, of which 135 were up-regulated and 120 were down-regulated (Figure S1D). The differentially acetylated proteins suggested that post-translational modification has a potential role in wheat seed ageing.

### 3.3. Functional Annotation, Enrichment and Protein-Protein Interaction (PPI) Network Analysis

Protein annotation was carried out to better elucidate the biological roles of the acetylated proteins. The results indicated that the up-regulated acetylated proteins were involved in cellular and metabolic processes and biological regulation, most of which were located in cell (32%), organelle (27%), macromolecular complex (20%) and the extracellular region (14%). Additionally, most of the up-regulated differential acetylated proteins were linked to binding and catalytic activity (Figure S2A–C). Proteins corresponding to down-regulated acetylation sites were involved in metabolic process, cellular process, single-organism process, response stimulus and other biological processes with 33% in cells, 20% in cell membranes, 20% in organelles and 20% in macromolecular complexes. In terms of molecular function classification, the down-regulated differential acetylated proteins function in catalytic activity, binding and antioxidant activity (Figure S2D–F).

**Figure 3:**
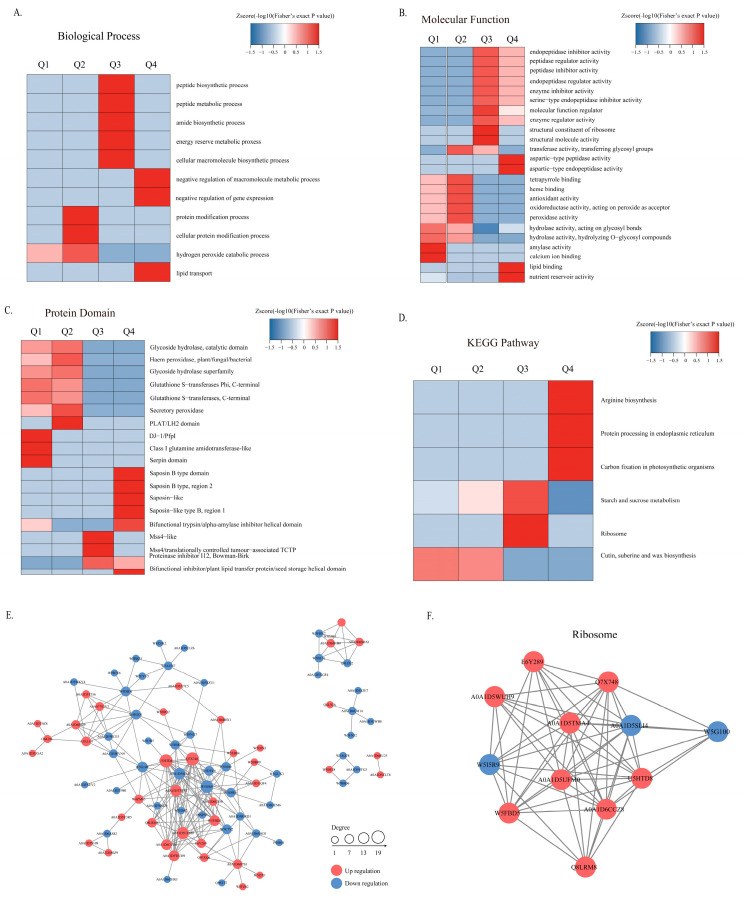
Functional enrichment analysis of Kac-modified proteins in terms of biological processes (**A**), molecular function (**B**), protein domain (**C**) and KEGG pathway (**D**). (**E**,**F**) Proteome interaction network.

Functional enrichment cluster analysis was carried out to better elucidate the function of acetylated proteins. Proteins with different modification levels were classified into four parts, namely, Q1 (0–0.67), Q2 (0.67–0.77), Q3 (1.3–1.5) and Q4 (>1.5), according to different ratios (Figure S2G). The GO enrichment analysis indicated that the down-regulated acetylated proteins (Q1 and Q2) were engaged in protein modification process, hydrogen peroxide catabolic process, antioxidant activity, peroxidase activity, hydrolase activity, amylase activity and calcium ion binding. Additionally, the up-regulated acetylated proteins (Q3 and Q4) participated in peptide metabolic process, energy reserve metabolic, negative regulation of gene expression, lipid transport, enzyme regulator activity, peptidase activity regulation, lipid binding and nutrient reservoir activity (Figure 3A,B). Protein domain cluster analysis showed that proteins related to glutathione-S-transferases, peroxidase, glutathione-S-transferases and secretory peroxidase were enriched in Q1 and Q2, whereas the proteins associated with saponin, plant lipid transfer protein, bifunctional inhibitor and/or seed storage were enriched in Q3 and Q4 (Figure 3C). KEGG analysis demonstrated that the down-regulated differential acetylated proteins were involved in cutin, suberine and wax biosynthesis, whereas the up-regulated acetylated proteins were associated with arginine biosynthesis, ribosome, protein processing in endoplasmic reticulum and starch and sucrose metabolism (Figure 3D). The results suggest that Kac might play a regulatory role in the ageing process of wheat by regulating enzyme activities in protein processing and detoxifying ROS, including glutathione-S-transferases and peroxidase.

The PPI networks of all YM6/YM97 differentially expressed acetylated proteins were mapped using the STRING database (http://www.ebi.ac.uk/string/8/8/2022 (accessed on 20 October 2022)) [22] and Cytoscape software 3.7.0 (Figure 3E). The results showed that the proteins in ribosomes were enriched. This finding was consistent with the KEGG clustering analysis, including ribosome and protein processing, and further supports the potential regulatory role of acetylation modification in ribosome activity (Figure 3F).

### 3.4. Categorisation of Representative Acetylated Proteins

Representative differentially expressed proteins (YM6 vs. YM97) were classified into five groups, namely, defense, signal transduction, metabolism, protein storage and processing and enzyme activity, to better uncover the functions of differentially expressed acetylated proteins (Table 1).

As expected, the modification level of enzymes involved in ROS detoxification (GST, monodehydroascorbate reductase and peroxidase) and stress response (serpin-N3.2, serpin-Z1C, serpin-Z1B, xylanase inhibitor protein 1, thaumatin-like protein, defensin-like protein, puroindoline b-like protein and caleosin) were remarkably down-regulated. In addition, the abundance of proteins related to heat shock response, such as heat shock protein, calmodulin and histidine-containing phosphotransfer protein, were also down-regulated. The results indicated that the resistance of seeds to high-temperature environment was reduced.

In the process of seed ageing, seed vigor and germination rate decreased, and the acetylation level of proteins involved in several metabolic pathways were varied. The modification levels of various enzymes related to glycolysis and TCA cycle, such as pyruvate kinase, citrate synthase, triosephosphate isomerase, pyruvate decarboxylase, malic enzyme, sucrose synthase and starch synthase, were all down-regulated. The modification levels of enzymes related to starch hydrolysis, such as α/β-amylase, decreased, whereas the modification levels of α-amylase/trypsin inhibitors CM2 and CM16 increased, which might be inconsistent with the changes in enzyme modification levels. Additionally, the modification levels of several storage proteins, such as γ-gliadin 1, globulin, oil body-associated protein 1A and cupincin, decreased, whereas the protein disulfide-isomerase (PDI) and aspartate aminotransferase modifications related to protein synthesis were up-regulated.

### 3.5. Effect of Acetylation Modification on GST Activity

**Figure 4:**
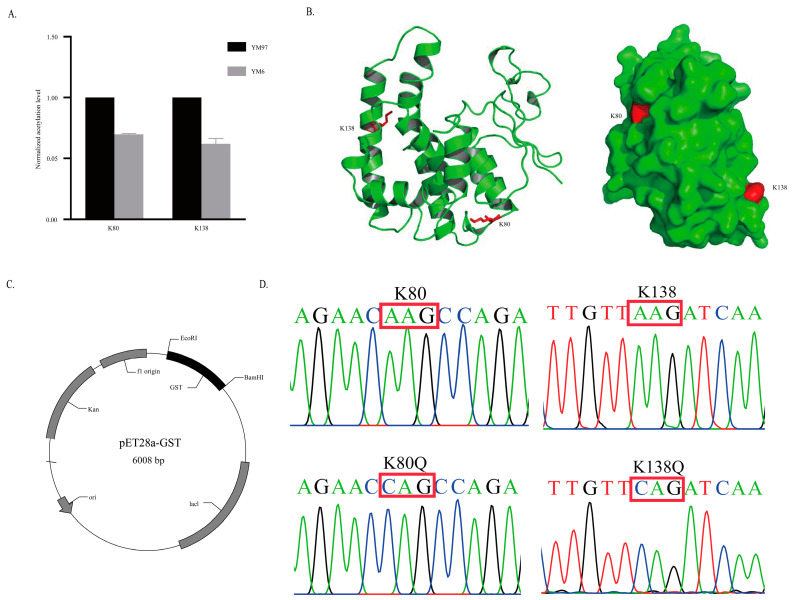
Structure of GST and Kac mutation simulation. (**A**) The acetylation level of GST K80 and K138 sites. (**B**) Structure and position of Kac-modified sites. (**C**) Construction of plasmid for GST expression. (**D**) Sequence analysis of site mutagenesis. The red box represents the mutation site base sequence.

**Figure 5:**
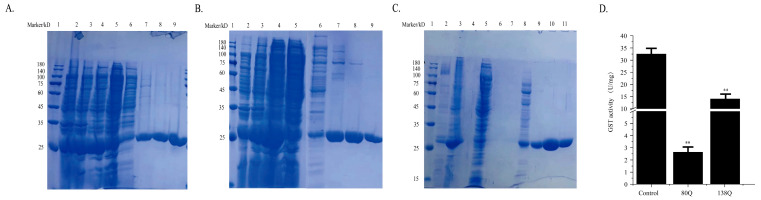
GST expression, purification and enzyme activity measurement. (**A**,**B**) GST and GST-80Q protein affinity chromatography purification. lane 1: Marker; lane 2: Pre-induction of GST and GST-80Q; lane 3: After-induction of GST and GST-80Q; lane 4: Supernatant; lane 5: Flow through, proteins without binding proteins are washed; lane 6–9: Different concentrations of imidazole wash. (**C**) GST-138Q protein affinity chromatography purification. lane 1: Marker; lane 2: Pre-induction of GST-138Q; lane 3: After-induction of GST-138Q; lane 4: Loading; lane 5: Flow through; lane 6–11: Different concentrations of imidazole wash. (**D**) Effect of Kac on GST activity. ** represents *p <* 0.001.

Differentially expressed lysine acetylated enzymes are likely to function in the ageing process. GST involved in detoxifying ROS was selected for verification to investigate the role of acetylation modification in wheat ageing process. The acetylation levels of K80 and K138 were significantly decreased in YM6 vs. YM97 (Figure 4A). The structure analysis showed that the acetylation sites were located on the surface and recombinant plasmid pET28a-GST was constructed for GST expression (Figure 4B,C). Then, the lysine at GST 80 and K138 sites were mutated to glutamine (Q) to simulate acetylation and the sequencing analysis demonstrated that the mutation was successfully introduced (Figure 4D). Then, GST was cloned, expressed and purified. GST protein was present in imidazole eluent at 20, 40 and 300 mM (Figure 5A–C). Then, the enzyme activities of GST and its mutants were measured with the unmutated GST as the control. The results showed that the enzyme activities were considerably decreased (Figure 5D). These results demonstrated that the presence of acetylation modification at sites K80 and K138 of GST might play a key role in GST activity and thus play an important role in the ageing process of wheat. 

## 4. Discussion

During long-term storage, the germination rate and vitality of seeds decreased, and the quality continued to decline. Previous studies indicated that enzymes that participate in the metabolic process are altered during the ageing process [10]. Recent reports also demonstrated that PTM is conserved in animals, plants and microorganisms and plays a vital role in metabolic regulation and response to intra- and extracellular environmental stresses [23]. Research indicated that PTM might serve an important role in the ageing process of grain seeds. Hence, quantitative acetylome was performed to artificially aged wheat seeds to investigate the effect of acetylation modification in the ageing process of wheat seeds, and the effect of acetylation on GST activity was studied by point mutation to elucidate its role in wheat seed ageing.

ROS plays important roles in seed ageing. Many enzymes in the cell eliminate this harmful factor, and GST is one of them [6]. A previous quantitative proteomic study demonstrated that defense-related proteins and GST involved in ROS detoxification are up-regulated during accelerated ageing in ‘Yangmai 15’ seeds [10]. GST is a large family encoded by multiple genes and widely exists in animals, plants and microorganisms [24,25]. It also plays a vital role in the detoxification of xenobiotics, herbicide, plant growth and development and response to biotic and abiotic stresses [25,26]. Song et al. [27] also proved that GST in insects is resistant to a variety of oxidative stress conditions, such as cold, heat, ultraviolet radiation and pathogenic microbes, whilst detoxifying a variety of insecticides, such as phoxim, dichlorvos and carbofuran. Recent study found that during wheat germination, GST has remarkably up-regulated acetylation levels at K42, K50, K188 and K145; the acetylation levels of these sites may contribute to the ability of GST to better cope with oxidative stress [28]. In the present study, the protein acetylation level and acetylation modification level at sites 80 and 138 of GST involved in stress response decreased considerably in YM6 versus YM97, suggesting that GST might play a certain resistance role in wheat ageing. Hence, we further carried out point mutations on these two sites of GST to mimic acetylation. The results showed that enzyme activities remarkably reduced. The results indicated that acetylation modification may lead to the conformational change of GST protein and attenuate the activity of GST protein, which is similar to the result of point mutation at K322 of isocitrate lyase, a key enzyme in the citrate cycle of Mycobacterium tuberculosis [29]. The decreased activity of GST modified by the Kac indicated that the resistance ability of wheat seeds to harsh environmental stress was gradually diminished, and its ageing process might be accelerated. Monodehydroascorbate reductase (MDHAR) and peroxidase also play an important role in the anti-ROS process. In plants, MDHAR can maintain the level of cellular ascorbate and thus play a protective role [30]. Peroxidase is a kind of protective enzyme, which can timely and effectively remove free radicals, control lipid peroxidation, reduce damage to the membrane system and ensure the normal growth and development of seeds [31]. In the present work, the modification level of MDHAR was also down-regulated, which might cause the loss of wheat seed vitality. In the current work, the abundances of three peroxidase enzymes (Table 1) were also down-regulated, which is consistent with the changes in ageing cotton seeds [31], and the altered acetylation level might be responsible for the attenuated resistance to environmental stress during wheat seed ageing process.

Several stress-related proteins change during artificial ageing to protect wheat from biotic and abiotic stresses. The expression of serpin family is considerably increased under abiotic stress conditions, such as salt and cold, which play a key role in plant growth and development and defense stress [32]. Xylanase inhibitor protein can prevent xylanase degradation to protect the cell wall and cope with water deficit stress [32]. Additionally, thaumatin-like proteins are a highly complex protein family that play a remarkable role in protection against biotic and abiotic stresses (drought, high salt, osmotic stress and oxidative stress) [33]. In the current study, the modification degree of stress-related proteins, such as serpin-N3.2, serpin-Z1C, serpin-Z1B, xylanase inhibitor protein 1, thaumatin-like protein and defensin-like protein, were down-regulated, which might lead to the weakened ability of wheat to resist adverse environmental conditions and then affected wheat ageing. Additionally, puroindoline is a unique antifungal membrane protein in wheat. Studies have shown that puroindolin B (PINB) exerts antifungal effects by disrupting the cell wall and membrane integrity of *Aspergillus flavus* and enzymes related to the activity of the cell membrane. Moreover, PINB could also aggravate the oxidative stress induced by *A. flavus* [34]. In our study, the abundance of modifications of puroindoline b-like protein was reduced, indicating that the antifungal activity of wheat was reduced, and its resistance to the external environment might be decreased. In *Arabidopsis thaliana*, caleosin affects lipid metabolism and responds to abiotic stress processes, such as ABA, drought, infiltration and salt [35]. In the present work, the modification levels of three caleosin proteins were down-regulated, suggesting stomata enlargement and water loss under the condition of artificial ageing.

Signal transduction pathway functions in the process of heat shock. Calmodulin is an important intermediate of calcium-mediated signaling and is involved in heat shock signaling in wheat [36]. Cytokinin is a growth regulator that affects cell division, bud differentiation and delay senescence, and histidine-containing phosphotransfer protein 2 plays a positive role in cytokinin signaling [37]. The results showed that acetylation levels of calmodulin and histidine-containing phosphotransfer protein were down-regulated in the process of ageing, in which calmodulin undergoes acetylation at the functional calcium binding site. The changes in these proteins may affect heat shock and cytokinin signal transduction and promote seed ageing. Likewise, heat shock 70 kD protein is also related to stress response. Its expression increases when cells are adversely affected by high temperature and pressure; thus, it better protects cells and regulates plant growth and development [38]. Previous studies showed that heat shock protein 70S is crucial to the heat tolerance, growth and development of the germinated seeds of *A. thaliana* [39]. Additionally, in rice and pepper, the expression of the heat shock protein gene is enhanced with the increase in temperature, and the accumulation of heat shock protein is closely related to plant heat stress response and heat tolerance [40,41]. This study found that the modification levels of two heat shock 70 kD proteins were also down-regulated, indicating that the tolerance of seeds to high-temperature conditions was changed.

Studies have shown that protein acetylation related to metabolism plays a prominent role in seed germination and seed vitality [42]. Nallamilli et al. found that glyceraldehyde-3-phosphate dehydrogenase and enolase, which participate in glycolysis. were acetylated in rice [43]. He et al. found that glyceraldehyde-3-phosphate dehydrogenase, pyruvate decarboxylate, ketoglutarate dehydrogenase, succinyl-coA synthase and other proteins related to glycolysis pathway and TCA cycle were up-regulated during rice seed germination and were of great importance for the recovery of seed carbon metabolism and energy supply [42]. Citrate synthase, a key enzyme involved in the TCA cycle and amino acid biosynthesis, is acetylated in *A. thaliana* [44] and *E. coli* [45]. Studies have shown that the mutation of lysine residue K322 of isocitrate lyase into glutamine to mimic acetylation could lead to a remarkable decrease in enzyme activity [29]. In the present study, the abundances of modifications linked to the glycolytic pathway and TCA cycle, such as pyruvate kinase, citrate synthase, triose phosphate isomerase, pyruvate decarboxylase and malic enzyme, were decreased. Additionally, the acetylation modification levels of sucrose synthase involved in the synthesis of storage materials and seed development [46] and starch synthase involved in starch synthesis were also down-regulated [47]. Our study found a higher proportion of proteins associated with binding and catalytic activity, suggesting that enzymes related to metabolism might be the major protein species for acetylation modification. A similar phenomenon was observed in rice and tea [21,48]. In human cells, the acetylation level of enzymes related to metabolism is influenced by the concentrations of glucose, amino acids and fatty acids and affects the activity and stability of enzymes [49]. The changes of these enzymes indicated that the related metabolic pathways involved in these proteins were interfered during seed ageing, which affected seed germination and seed vitality and then led to the decrease of germination rate.

Storage proteins accumulate during wheat seed development, provide nutrients for germination and decrease with seed ageing. Glutenin and gliadin account for a large proportion of wheat protein in starchy endosperm cells and affect wheat grain quality and processing quality [50]. Storage proteins are nitrogen sources required for seed germination; these proteins are hydrolyzed by proteases to provide amino acids required for seed germination [51]. In our work, the degrees of acetylation modification of gamma-gliadin 1, glutenin, globulin, oil body-associated protein 1A and cupincin were down-regulated. Therefore, the down-regulation of the modification degree of these storage proteins might result in conformational changes that ultimately lead to the insufficient supply of energy and nutrient for seed germination, resulting in decreased seed vitality.

During the process of wheat ageing, storage proteins are processed by ribosomes and the endoplasmic reticulum. KEGG cluster analysis showed that the up-regulated differentially modified proteins were related to ribosomes and protein processing in the endoplasmic reticulum. PDI catalyzes the formation of intramolecular disulfide bonds in storage proteins in the rough endoplasmic reticulum, assists in the folding of newly synthesized hydrolases during grain development and has special properties in determining bread-making quality and flour technological properties [52]. In this study, the level of PDI acetylation modification was up-regulated, suggesting that the protein structure might change and affect protein processing and wheat germination. Moreover, aspartic acid is associated with protein synthesis, provides nitrogen for growth and is vital for seed germination [53]. In our study, the abundance of aspartate aminotransferase was up-regulated, which might lead to a change in enzyme activity and affect the germination vitality of seeds. Hydrolytic enzymes are activated to decompose intracellular nutrients and generate energy during seed germination. During seed germination, the activity of α-amylase increases, which promotes the hydrolysis and transformation of internal starch and provides energy for germination [53]. In the present work, the modification level of α-amylase was down-regulated, and those of α-amylase/trypsin inhibitor CM2 and CM16 were up-regulated. A previous study found that the site-directed mutations of Asn329 by Lys and Asp331 by Glu of α-amylase in *Bacillus thermophilus* resulted in an almost complete loss of enzyme activity, and the mutation of Hi338 to Asp reduced enzyme stability [54,55]. In the present study, acetylation modification occurred at site 208 of α-amylase. The activity and stability of the enzyme may also be affected to some extent. Furthermore, α-amylase/trypsin inhibitors are beneficial to plants, as they resist injury and promote organ development [56]. Chemical modification of the amino group in the α-amylase inhibitor molecule of cranberry bean causes a rapid loss of inhibitory activity [57]. The down-regulation of α-amylase/trypsin inhibitor modification levels was found in the present study. This finding suggests that the acetylation modification could inhibit the activities of α-amylase/trypsin inhibitors and is not conducive for wheat seeds to resist damage from the external environment; thus, wheat grain quality might be adversely affected.

In summary, we reported a study on the regulation of wheat grain ageing by acetylation modification. Acetylated proteins are involved in various biological processes, especially metabolic and defensive stress responses, indicating that artificial ageing influenced these processes. Moreover, point mutation results showed that the acetylation modification of GST K80 and K138 affected its enzyme activity, which in turn played a role in seed ageing. Our results help elucidate the potential regulatory role of acetylation modification in wheat ageing and further deepen our understanding of the mechanism during wheat seed storage.

## Figures and Tables

**Table 1:** Classification of acetylated differential proteins in YM6 vs. YM97.

Protein Accession	Protein Description	Regulated Type
Defense		
Q9SP56	Glutathione S-transferase	down
0A1D5Y508	Monodehydroascorbate reductase	down
A0A1D5WFB5	Peroxidase	down
A0A1D6D528	Peroxidase	down
A0A1D5X0A7	Peroxidase	down
Q9ST57	Serpin-N3.2	down
Q9ST58	Serpin-Z1C	down
W5FZ62	Serpin-Z1B	down
Q8L5C6	Xylanase inhibitor protein 1	down
A0A1D5XU85	Xylanase inhibitor protein 1	down
W5FN32	Thaumatin-like protein	down
P20158	Defensin-like protein 1	down
A0A1D6S4Q3	Defensin-like protein 1	down
A0A1D6S4Q2	Defensin-like protein 2	down
B1GXL6	Puroindoline b-like protein 2v2	down
W5AQ78	Caleosin	down
W5B8D6	Caleosin	down
A0A1B5GE57	Caleosin	down
W5E0J0	Heat shock 70 kDa protein	down
W5DYF8	Heat shock cognate 70 kDa protein	down
Signal transduction		
P04464	Calmodulin	down
I7LRG9	Histidine-containing phosphotransfer protein 2	down
Metabolism		
W5G100	Pyruvate kinase	down
W5H1Q1	Citrate synthase	down
W5DIU6	Triosephosphate isomerase	down
A0A1D5XEV2	Pyruvate decarboxylase 2	down
A0A1D5WCE5	Malic enzyme	down
W5B5R3	Sucrose synthase	down
Q9LEE2	Starch synthase, chloroplastic/amyloplastic	down
Protein storage and processing		
M9TG60	Gamma-gliadin 1	down
A0A1D5SVW4	Glutenin, high molecular weight subunit	down
A0A1D5SEL0	Basic 7S globulin	down
A0A1D5YFG3	Oil body-associated protein 1A	down
A0A1D5YEH1	Cupincin	down
A0A1D5X7C5	Protein disulfide-isomerase	up
Enzyme activity		
A0A1D5XGF4	Beta-amylase	down
W5FHN7	Alpha-amylase	down
A0A1D5UB33	Alpha-amylase/trypsin inhibitor CM2	up
P16159	Alpha-amylase/trypsin inhibitor CM16	up
A0A1D6ACI6	Alpha-amylase/trypsin inhibitor	down
A0A077S3V2	Aspartate aminotransferase	up

## Data Availability

The mass spectrometry proteomics data have been deposited to the ProteomeXchange Consortium via the PRIDE partner repository with the dataset identifier PXD034134.

## Supplementary Materials

The following supporting information can be downloaded at: https://www.mdpi.com/article/10.3390/foods11223611/s1. Figure S1: Quality control of the peptides and overview of the acetylome; Figure S2: GO annotation of the acetylated proteins and category of the differentially acetylated sites.
