# Supplementary material for: Quantitative Acetylome Analysis of Soft Wheat Seeds during Artificial Ageing
Source: Foods. 2022 Nov 12;11(22):3611. doi: 10.3390/foods11223611 (PMC9689531; doi:10.3390/foods11223611)
Supplement: Supplementary file 1 [file foods-11-03611-s001.zip › foods-2014411-supplementary.pdf]

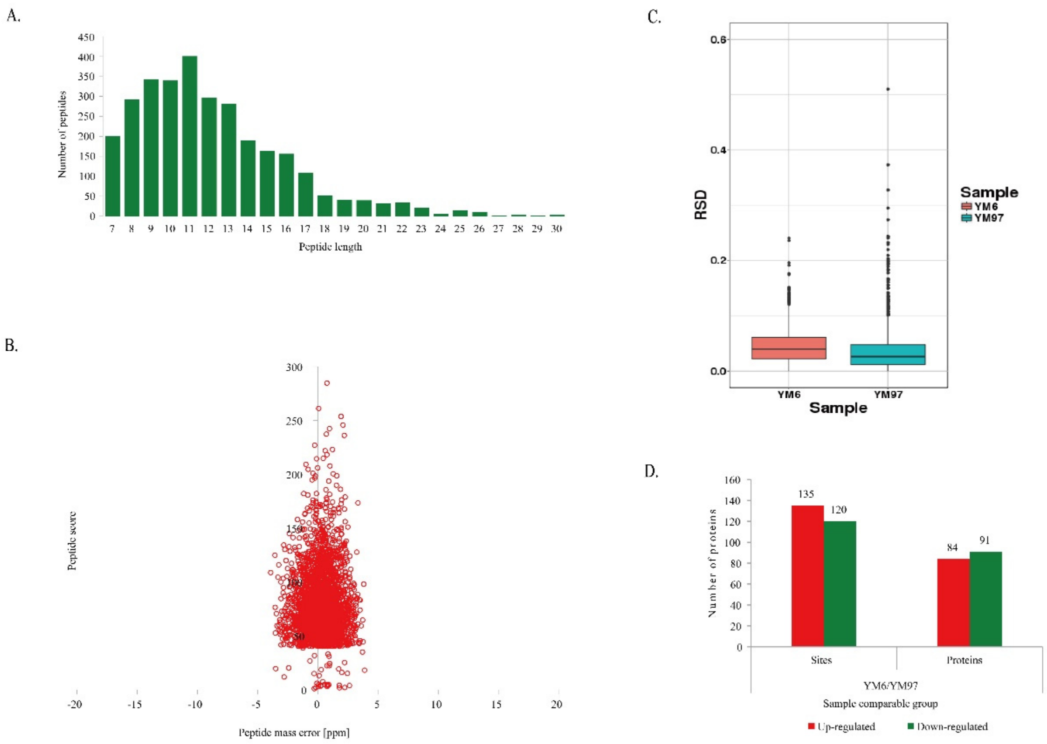

Figure S1. Quality control of the peptides and overview of the acetylome. (A) Peptide length distribution; (B) mass offset distribution of the peptide; (C) box plot with standard deviation coefficient (RSD) between repeated samples; (D) differential acetylated modified proteins and number of modified sites.

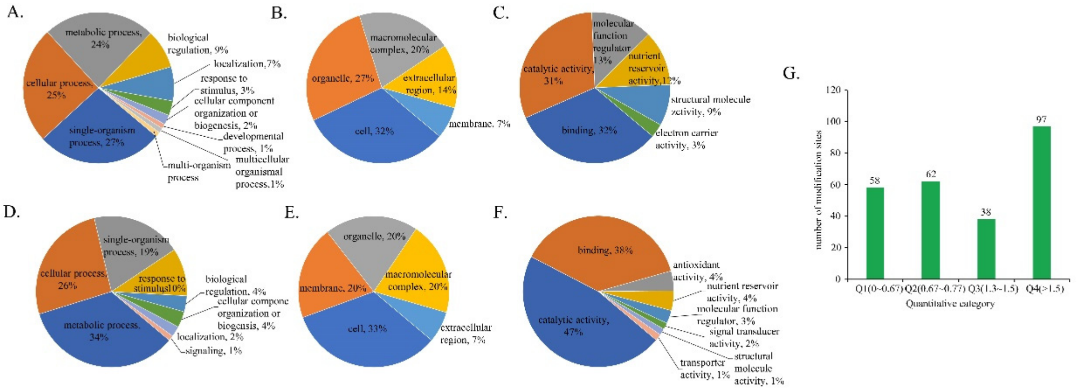

Figure S2. GO annotation of the acetylated proteins and category of the differentially acetylated sites. GO annotation (YM6/YM97) of up-regulated modifying proteins in (A) biological processes, (B) cellular components and (C) molecular function and down-regulated modifying proteins in (D) biological processes, (E) cellular component (F) and molecular function; (G). Number of differential proteins at different differential multiples.
